# Hypoglycemia Secondary to Sulfonylurea Ingestion in a Patient with End Stage Renal Disease: Results from a 72-Hour Fast

**DOI:** 10.1155/2015/742781

**Published:** 2015-11-17

**Authors:** Alice Abraham, Mishaela Rubin, Domenico Accili, John P. Bilezikian, Utpal B. Pajvani

**Affiliations:** Division of Endocrinology, Department of Medicine, Columbia University College of Physicians and Surgeons, New York, NY 10032, USA

## Abstract

Insulin, proinsulin, and C-peptide levels increase with sulfonylurea exposure but the acuity of increase has not been described in dialysis patients. We present a case of a dialysis patient who presented with hypoglycemia and was found to have accidental sulfonylurea ingestion. This is a 73-year-old man with ESRD on peritoneal dialysis, without history of diabetes, who presented with hypoglycemia. Past medical history includes multiple myeloma, congestive heart failure, and hypertension. At initial presentation, his blood glucose was 47 mg/dL, with concomitant elevations in the following: C-peptide 30.5 (nl: 0.8–3.5 ng/mL), insulin 76 (nl: 3–19 *μ*IU/mL), and proinsulin 83.3 (nl: ≤8.0 pmol/L). During the 72-hour fast, which he completed without hypoglycemia, insulin declined to be within normal limits (to 12 *μ*IU/mL); proinsulin (to 12.1 pmol/L) and C-peptide (to 7.2 ng/mL) levels decreased but remained elevated. The sulfonylurea screen ultimately returned positive for glipizide, clinching the diagnosis. This is the first reported case which characterizes the chronic elevation of proinsulin in a patient with ESRD, as well as its dramatic increase after a presumed solitary exposure to sulfonylurea. The 72-hour fast conducted gives insight into the clearance of insulin, proinsulin, and C-peptide after sulfonylurea ingestion in ESRD.

## 1. Introduction

Insulin, proinsulin, and C-peptide levels increase with sulfonylurea exposure but the degree and acuity of increase are not known in dialysis patients. We describe a man with end stage renal disease (ESRD) on peritoneal dialysis with no previous history of diabetes who presented with unexplained hypoglycemia. To determine the source of his protracted hypoglycemia, he underwent a 72-hour fast in which we observed progressive declines in insulin, proinsulin, and C-peptide levels. Sulfonylurea screen returned positive for glipizide. The 72-hour fast gives insight into the rate of C-peptide, proinsulin, and insulin clearance in ESRD after sulfonylurea ingestion, which, to our knowledge, has not yet been described in the literature.

## 2. Case Report

We describe a 73-year-old man with ESRD on peritoneal dialysis, without history of diabetes, who presented to the emergency department (ED) with hypoglycemia. Past medical history was notable for multiple myeloma, congestive heart failure, and hypertension. His ESRD was the result of longstanding hypertension and was diagnosed years before the multiple myeloma. Nine months prior to presentation, the patient received three cycles of thalidomide for multiple myeloma in his native Dominican Republic, but no recent or new chemotherapeutic agents were added prior to admission. In the ED, he was diaphoretic and found to have fingerstick glucose of 43 mg/dL; his symptoms resolved immediately upon normalization of blood glucose. His hypoglycemia was recurrent with fingerstick glucose persistently in the 30–40 mg/dL range prior to initiation of a dextrose 10% (D10) drip. He required a D10 infusion for forty-eight hours before it could be safely titrated off.

The patient denied prior neuroglycopenic symptoms. He denied recent changes to his dialysate or symptoms of infection or adrenal insufficiency. His medications include a beta-adrenergic blocker, folic acid, nephrovite vitamin B complex, and omeprazole. Two weeks prior to presentation, his beta-adrenergic blocker dosage was increased; otherwise there were no changes to his medication regimen. He lives with two family members who both have Type 2 Diabetes on sulfonylurea (glipizide), but he denied accidental or intentional ingestion. His family members were unable to recall the dose and frequency of their glipizide medications. The patient performs nightly peritoneal dialysis. Throughout his hospitalization, he performed peritoneal dialysis using 1.5/2.5% Dianeal PD solution every 6 hours with a 2 L fill.

Physical exam was notable for normal vital signs [blood pressure 113/74, pulse 64, temperature 37.2 degrees Celsius, and respiratory rate 20]. He was overweight with a BMI of 26.6 kg/m^2^. He was a well appearing Hispanic elderly gentleman with acanthosis nigricans present over his posterior neck. Serum electrolytes were within normal limits (potassium 4.5 mmol/L [3.6–5.0], sodium 138.0 mmol/L [136–146], and chloride 101 mmol/L [97–107]). Creatinine and blood urea nitrogen (BUN) concentrations were both elevated at 10.00 mg/dL [0.60–1.20 mg/dL] and 48 mg/dL [7–20 mg/dL], respectively, but unchanged from baseline values (creatinine 9.48–12.82 mg/dL and BUN 48–83 mg/dL). The serum albumin concentration was mildly reduced at 3.1 [3.5–5.5 g/dL], attributed to malnutrition and chronic inflammation. The INR 1.1 [0.9–1.2] and transaminases (AST 15 [12–38 U/L] and ALT 11 [7–41 U/L]) were normal.

The hypoglycemia prompted measurement of serum fructosamine which was normal, 186 [170–285 umol/L], but interpretation is potentially confounded by his low albumin and multiple myeloma history. Similarly, the hemoglobin A1C was normal at 5.7 but was also difficult to interpret in ESRD. Additional laboratory evaluation included normal cosyntropin stimulation testing (36.7 *μ*g/dL before cosyntropin to 42.7 *μ*g/dL 1 hour after cosyntropin administration) and negative infectious disease workup, including urine, blood, and peritoneal dialysate fluid cultures. CT scan of the abdomen with intravenous contrast performed six months prior to admission for abdominal pain revealed a normal pancreas.

At the time of admission, the blood glucose was 47 mg/dL, with concomitant elevations in the following: C-peptide 30.5 (nl: 0.8–3.5 ng/mL), insulin 76 (nl: 3–19 *μ*IU/mL), and proinsulin 83.3 (nl: ≤8.0 pmol/L). C-peptide, insulin, and proinsulin were measured by ARUP laboratories using quantitative chemiluminescent immunoassays. Our differential diagnosis for hyperinsulinemic hypoglycemia included insulinoma versus exposure to an oral hypoglycemic agent. We entertained the possibility of an insulin antibody mediated hypoglycemia in light of his multiple myeloma history [1]; however, insulin antibody was not detectable. As it would take days to receive the results from his sulfonylurea screen and we could not entirely eliminate insulinoma from the differential, he underwent a 72-hour fast to determine whether the source of the hypoglycemia was exogenous or endogenous.

**Figure 1:**
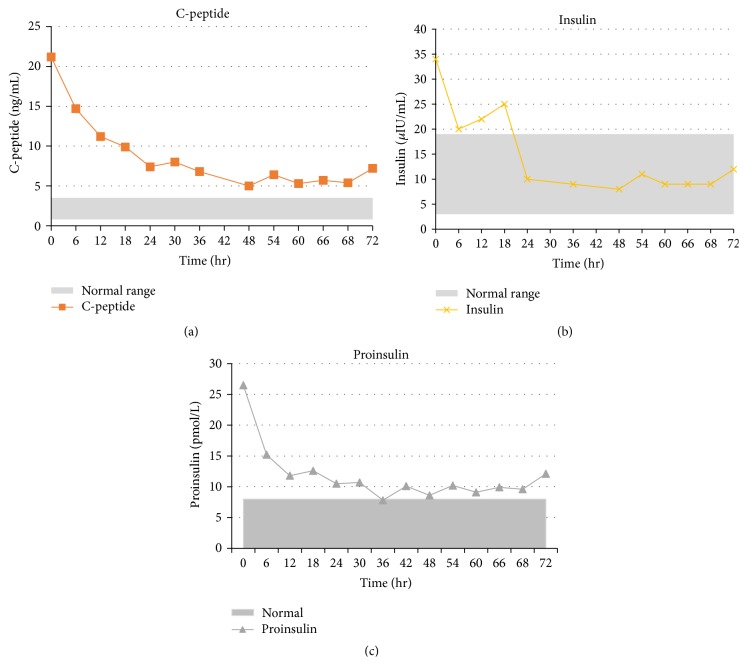
Decline of C-peptide, insulin, and proinsulin observed during 72-hour fast.

During the 72-hour fast, insulin levels rapidly declined within 24 hours to normal; proinsulin and C-peptide levels fell in parallel but remained abnormally high (Table 1 and Figure 1). The patient completed the fast without developing further hypoglycemia or neuroglycopenic symptoms. The sulfonylurea screen, obtained at the time of admission, was positive for glipizide (performed by ARUP laboratories, positive at concentrations greater than 5 ng/mL). The patient and family denied inadvertent use of glipizide. The patient was discharged with new prescriptions in case there was a pharmacy error. When the patient returned for outpatient laboratory testing 1 week later, the repeated values were similar to those obtained at the end of the fast, with persistently elevated fasting C-peptide (8.8 ng/mL) and proinsulin (10.7 pmol/L) but normal insulin (14 *μ*IU/mL) levels. He denied any recurrent hypoglycemia symptoms one week after hospital discharge. Furthermore, during subsequent admissions for worsening congestive heart failure, he exhibited no further signs or symptoms of hypoglycemia.

## 3. Discussion

Sulfonylurea ingestion is a well-recognized cause of hyperinsulinemic hypoglycemia. In this case, we believe this was our patient's first exposure to sulfonylurea, as he had no neuroglycopenic symptoms until shortly prior to admission. Although some medications such as H2 receptor blockers, salicylates, and trimethoprim-sulfamethoxazole are known to prolong the hypoglycemic effects of sulfonylureas [2, 3], this patient was not taking any of them. Thus, the data may well represent rates of recovery in proinsulin, C-peptide, and insulin levels after endogenous stimulation by sulfonylurea ingestion in renal failure.

Glipizide is a potent, second generation sulfonylurea, and, in patients with normal renal and liver function, it has an elimination half-time of about 7 hours and duration of action of 12 to 24 hours. Glipizide undergoes extensive enterohepatic metabolism and is converted in the liver to several inactive metabolites. Thus, in patients with cirrhosis, glipizide has a prolonged half-life [4]. Less than 5 percent is excreted in the urine as intact glipizide, with the majority excreted as inactive metabolites (60 percent as 4-hydroxyglipizide, 15 percent as 3-hydroxyglipizide, and 2 percent as N-(*β*-acetyl-aminoethyl-benzene-sulfonyl)-N-cyclohexyl-urea) [5]. Unlike sulfonylureas such as acetohexamide and glyburide which are metabolized to active metabolites [6], one would not expect accumulation of glipizide metabolites to have a significant clinical impact in renal insufficiency. In fact, the package insert for glipizide suggests caution but no dose adjustment in renal impairment. Some, however, have recommended a 50% reduction in dose in patients with GFR ≤ 50 mL/minute [7].

The biochemical markers of endogenous hyperinsulinism documented in this case suggest unique rates of disappearance of insulin and its precursors in patients with ESRD. We found a marked relative increase in proinsulin and C-peptide (8–10x ULN) as compared to insulin (4x ULN) levels. Assuming an equivalent production rate, the disproportionate elevation of insulin precursors is most likely related to the relatively faster clearance of insulin (60–70% by liver, with the remainder by other insulin-responsive tissues) and proinsulin/C-peptide (by kidneys) [8, 9]. This estimate is substantiated by the progressive reduction in insulin levels to normal, whereas proinsulin and C-peptide levels declined but remained high, even after completion of the 72-hour fast (Table 1 and Figure 1). Elevations of C-peptide in renal insufficiency are thought secondary to impaired renal degradation and excretion into urine, exacerbated by insulin resistance induced by uremia [9]; the effects of renal failure on proinsulin are known to be similar [10, 11] although the mechanism was not as clearly understood.

One of the more surprising aspects of this case is the rapid and marked increase in proinsulin in response to acute sulfonylurea exposure. Proinsulin is synthesized in the endoplasmic reticulum of beta cells and then cleaved to mature insulin and C-peptide prior to packaging into secretory vesicles [12]. It is known that, in Type 2 Diabetes (T2D), proinsulin levels are mildly elevated and that there is a further 30% increase with chronic sulfonylurea therapy [13], but why this precursor appears in serum at all is unclear. Two theories are worth considering: (1) excessive secretory demand of beta cells and (2) impaired proinsulin processing in the face of chronic hyperglycemia in T2D patients. Our results suggest that even after acute exposure in a nondiabetic patient, proinsulin levels can rise by 3-fold over baseline, suggesting that hyperproinsulinemia in response to sulfonylureas is likely caused by excessive secretory demand of an “unprepared” beta cell. In this patient, hyperproinsulinemia was likely further enhanced by an element of insulin resistance, as evidenced by acanthosis nigricans on the physical examination. Thus, he may have been “primed” by underlying insulin resistance and baseline chronic elevation of his insulin secretion machinery which together exacerbated sulfonylurea-induced hypoglycemia. One of the unavoidable limitations of this case was the fact that the patient's peritoneal dialysis solution contained a low amount of glucose, but this does not invalidate the results described.

Although C-peptide levels are known to increase with ESRD and sulfonylurea therapy, to our knowledge, this is the first reported case which characterizes the acute and chronic increase in proinsulin after a first and presumed solitary exposure to a sulfonylurea in an ESRD patient.

## Figures and Tables

**Table 1:** Results of C-peptide, proinsulin, insulin, and glucose obtained during 72-hour fast.

Time (hours)	C-peptide (normal 0.8–3.5 ng/mL)	Proinsulin (normal ≤ 8 pmol/L)	Insulin (normal 3–19 *μ*IU/mL)	Glucose (mg/dL)
0	21.2	26.5	34	115
6	14.7	15.2	20	101
12	11.2	11.8	22	104
18	9.9	12.6	25	107
24	7.4	10.5	10	108
30	8	10.7		104
36	6.8	7.8	9	100
42		10.1		90
48	5	8.6	8	80
54	6.4	10.2	11	95
60	5.3	9.1	9	82
66	5.7	9.9	9	106
68	5.4	9.6	9	67
72	7.2	12.1	12	95
